# Noninvasive Ventilation in Pediatric Bronchiolitis: Variables Related to Weaning Readiness and Approach

**DOI:** 10.1155/carj/2042835

**Published:** 2026-08-20

**Authors:** Rheanna Bulten, Gregory Hansen, Tanya Holt

**Affiliations:** ^1^ Department of Pediatrics, University of Saskatchewan, Saskatoon, Saskatchewan, Canada, usask.ca; ^2^ Pediatric Intensive Care, Jim Pattison Children’s Hospital, Saskatoon, Saskatchewan, Canada

**Keywords:** bronchiolitis, critical care, infant, noninvasive ventilation, ventilator weaning

## Abstract

**Background:**

There is a lack of evidence related to weaning noninvasive ventilation in bronchiolitis. The purpose of this study was to identify variables related to NIV weaning readiness and approach in bronchiolitis.

**Methods:**

A retrospective cohort study of 74 children less than two years of age with bronchiolitis, requiring NIV and admitted to a pediatric intensive care unit (PICU) from 2022 to 2023. Demographic variables, clinical metrics, NIV mode, and weaning metrics were collected.

**Results:**

Mean total duration of NIV therapy was 40 h with weans lasting 13 h. Duration for children on CPAP alone was shorter (26 h) than for children on combination of BiPAP and CPAP (45 h, 95% CI 4.258 to 33.742, *p* = 0.0123). Wean times were also shorter for children on CPAP alone (mean 5 h) than children on BiPAP and CPAP (mean 16 h) (95% CI 1.899 to 20.101, *p* = 0.0185). Patients were more likely to have a “complex wean,” defined as escalation and de‐escalation between multiple settings, with BiPAP (44.6%) than with CPAP (18.8%). RR at first wean was a significant predictor of NIV weaning duration (*p* = 0.007).

**Conclusion:**

There is a need for NIV weaning guidelines. RR may be an important predictor of NIV weaning readiness and approach. A “complex wean” may be a potential outcome variable for weaning NIV in children with bronchiolitis. This study may be used to inform future research and guidelines for weaning NIV in children with bronchiolitis.

## 1. Introduction

Bronchiolitis is the most common cause of hospitalization for children less than 2 years [1, 2]. In severe cases, noninvasive ventilation (NIV) has gained significant footing in pediatric intensive care units (PICUs) to provide respiratory support without the need for intubation [3–6]. Despite its increased use, there are no evidence‐based pediatric protocols for weaning. Weaning efficiency is important because NIV has been associated with numerous risks including aspiration, barotrauma, reduced preload, and skin and mucosal breakdown [7].

In children with bronchiolitis, a step‐down approach to weaning may lead to an increased duration of NIV [8], while weaning strategies using high‐flow nasal cannula for de‐escalation of NIV may lead to shorter PICU stays [9]. Surveys of weaning practices have shown nonuniformity [10]. Notably, there are very limited studies identifying weaning protocols for NIV. There is also large variability in weaning de‐escalation signals. Studies have identified respiratory rate (RR), wheeze, and retractions as markers of disease severity [11]. Other studies have suggested that apneas, the degree of respiratory distress, RR, hemodynamic status, and oxygen need may prompt NIV weaning initiation and identify failure [10, 12]. Clinical scoring tools such as the pediatric logistic organ dysfunction (PELOD) may be indicators of disease severity [13] but have been scarcely used in the context of weaning NIV in bronchiolitis. In 2024, a consensus panel reported high agreement for level of oxygenation, RR, and signs of respiratory distress as being fundamental measures for weaning NIV. However, the panel agreed evidence‐based criteria remained scarce, and future studies investigating weaning NIV in children are urgently needed [14].

In summary, there is a dearth of evidence for weaning NIV in children with bronchiolitis, and no uniform variables have been identified as triggers to initiate weaning. While expert consensus suggests that the weaning process should be standardized [14], opinions are not consolidated on how it should happen [10]. The primary purpose of this retrospective cohort study was to identify variables related to NIV weaning readiness in bronchiolitis and to explore how the weaning approach relates to weaning success. These results may help inform prospective research on developing an evidence‐based weaning protocol of NIV in bronchiolitis.

## 2. Methods

### 2.1. Variables

This retrospective study was approved by the University of Saskatchewan’s Research Ethics Board, and the study received exemption from informed consent given its retrospective and anonymous data (HREB 4103). Pediatric patients were identified through the Saskatchewan PICU’s admission registry. Inclusion criteria were as follows: (i) all pediatric patients under the age of 24 months; (ii) admitted to the Jim Pattison Children’s Hospital (JPCH) PICU between July 1, 2022, and July 1, 2023; (iii) with a diagnosis of bronchiolitis; and (iv) managed with NIV. In Saskatchewan, all children requiring NIV are managed in the PICU. The exclusion criteria were children with (i) any cardiac disease, including cardiac anomalies and previous cardiac surgery; (ii) chronic lung disease and/or on home oxygen; (iii) anatomic airway abnormalities; (iv) an immunodeficiency; (v) apnea; (vi) neuromuscular disease; (vii) born < 32 weeks’ gestation (extreme prematurity); and (viii) children requiring conventional mechanical ventilation (CMV).

All children included in this study tested positive by nasopharyngeal polymerase chain reaction for a viral pathogen. *Bronchiolitis* was defined as a viral respiratory tract infection causing swelling and irritation of the bronchioles. *NIV* included the use of continuous positive pressure ventilation (CPAP) and/or bilevel positive airway pressure (BiPAP). The term *complex NIV wean* emerged following data analysis and was defined as an NIV weaning course that included at least one of the following: (1) an escalation of NIV settings (e.g., an increase in expiratory PAP [EPAP], inspiratory PAP [IPAP], or FiO_2_), (2) an escalation of NIV modalities (e.g., CPAP to BiPAP), or both.

A pediatric resident (R.B.) and pediatric intensivist (T.H.) conducted all the electronic chart reviews. Data were stored on a password‐protected Excel file in a secure cloud. Variables included age, prematurity, sex, weight, respiratory virus(es) isolated, comorbidities, antibiotic, secondary bacterial infection, oxygen saturation (SpO_2_), RR, fraction of inspired oxygen (FiO_2_), C‐reactive protein (CRP), complete blood count (CBC), PELOD‐2 score on admission, and blood gas results (i.e., blood pH, hemoglobin, pCO_2_, and HCO_3_ values).

NIV data included settings (mode; cm H_2_O) at admission, maximum, and during weaning, highest FiO_2_, total duration on NIV, highest NIV support (cm H_2_O), FiO_2_ and NIV setting when wean began, duration of wean, and frequency of complex weans. Duration of weaning more specifically referred to the time in hours from the first weaning action (e.g., IPAP wean and EPAP wean) until a child was taken off and remained off NIV for the duration of the hospital stay. Outcomes included PICU length of stay (LOS) in days, hospital LOS in days, PICU days to low‐flow oxygen in days, 7‐day readmission rate, 7‐day ED visit rate, 30‐day readmission rate, 30‐day ED visit rate, and failure of rescue NIV (defined as going from NIV to CMV).

### 2.2. Statistics

All statistical analysis was completed using SPSS for Windows, Version 24. All data were pooled, and unique identifiers were removed. Discrete variables were reported as percentages, and continuous data were reported means and standard deviations. Descriptive statistics were used to compare clinical features and characteristics of PICU patients. The difference of means or Fisher’s exact test was used for further data analysis. Statistical significance was considered established at an alpha of 0.05.

## 3. Results

Seventy‐four children met inclusion criteria and are summarized in Table 1.

**TABLE 1:** Demographics and admission data (*n* = 74).

Variable	
Age (days), mean (SD)	189.2 (202.5)
Sex (male), *n* (%)	45 (60.8)
Weight (kg), mean (SD)	7.27 (3.22)
Comorbidities *n* (%)	10 (13.5)
RSV status,^1^ *n* (%)	44 (59.5)
Two or more viruses,^1^ *n* (%)	11 (14.9)
Other viruses^1^ *n* (%)	
Rhinovirus	12 (17.6)
Influenza A	1 (1.4)
COVID‐19	1 (1.4)
Human metapneumovirus	12 (16.2)
Adenovirus	4 (5.4)
Seasonal coronavirus	1 (1.4)
Parainfluenza	1 (1.4)
Secondary bacterial infection,^2^ *n* (%)	32 (43.2)
SpO_2_ (%), (SD)	95.7 (2.5)
Respiratory rate, (SD)	43.5 (13.1)
Heart rate, (SD)	160.6 (25.4)
pH, (SD)	7.33 (0.1)
HCO_3_‐ (mmol/L), (SD)	24.89 (4.34)
PCO_2_ (mmHg), (SD)	48.01 (11.6)
Lactate (mmol/L), (SD)	2.14 (1.2)
Hemoglobin (g/L), (SD)	116.96 (17.1)
Day of illness, (SD)	3.95 (1.7)
C‐reactive protein (mg/L), S	52.3 (56.6)
With bacterial infection	74 (67.8)
Without bacterial infection	32.6 (31.9)
Difference of square	*p* = 0.0008
White blood cell count (× 10^9^ cells/L)	11.1 (4.4)
Antibiotics, *n* (%)	50 (67.6)

*Note:* HCO_3_‐,venous bicarbonate; PCO_2_, venous partial pressure carbon dioxide; SpO_2_, peripheral oxygen saturation.

Abbreviations: RSV, respiratory syncytial virus; SD, standard deviation.

^1^Confirmed on laboratory testing.

^2^Urinary tract infections or bacteremia confirmed on laboratory testing.

Most children identified in our study were under the age of 12 months. All admission PELOD2 scores were between 0 and 1. The majority (59.5%) were infected with RSV, and nearly 15% had two or more viruses isolated. Forty‐three percent had a confirmed secondary bacterial infection, and 68% were treated with antibiotics. Those with confirmed bacterial infections had significantly higher CRP values (68 versus 32%; *p* value 0.0008).

NIV data are presented in Table 2. Children receiving BiPAP required a significantly longer duration of support (45.0 versus 26.0 h; *p* = 0.01) and wean time (16.0 versus 5.0 h; *p* = 0.02), had higher maximal FiO_2_ (38.0 versus 32.9%; *p* = 0.04), were more tachypneic at first wean (46.7 versus 39.3 RR; *p* = 0.01), and subjected to more complex weans (44.6 versus 18.8%; *p* < 0.05). No patient required escalation to mechanical ventilation. Specific to BiPAP weaning, the first wean occurred with IPAP, EPAP, or both levels simultaneously in 46.8% (*n* = 26), 5.4% (*n* = 3) and 48.2% (*n* = 27) of children, respectively. The first mean IPAP wean was 9.0 cm H_2_0 (SD 7.4), while the first mean EPAP wean was 3.1 cm H_2_0 (SD 2.3). In five instances (8.9%), BiPAP was initially weaned off NIV support. However, nearly all (*n* = 4; 80%) experienced complex weans. In total, 44.6% (*n* = 25) of children experienced complex weans.

**TABLE 2:** Noninvasive ventilation data.

Variable	NIV total (*n* = 74)	CPAP (*n* = 18)	BiPAP (*n* = 56)	CPAP vs. BiPAP (*p* value)
Duration (h), SD	40.0 (28.4)	26.0 (20.6)	45.0 (29.0)	0.01
Max setting (h), SD	27.3 (20.5)	20.6 (17.4)	29.4 (21.0)	0.07
Wean (h), SD	13.0 (20.0)	5.0 (12.9)	16.0 (21.5)	0.02
Max FiO_2_%, SD	36.4 (10.9)	32.9 (8.3)	38.0 (11.4)	0.04
First wean FiO_2_%, SD	28.3 (7.5)	28.6 (6.5)	28.3 (7.9)	0.87
First wean SpO_2_%, SD	94.7 (2.4)	94.6 (2.5)	95.1 (2.4)	0.49
First wean RR, SD	43.5 (12.3)	39.3 (11.3)	46.7 (12.4)	0.01
Complex wean total, *n* (%)	28 (38.0)	3 (18.8)	25 (44.6)	< 0.05

*Note:* BiPAP, bilevel positive airway pressure; FiO_2_, fraction of inspired oxygen; NIV, noninvasive ventilation; SpO_2_, peripheral oxygen saturation.

Abbreviations: CPAP, continuous positive airway pressure; RR, respiratory rate; SD, standard deviation.

Several variables were investigated as possible predictors of NIV wean duration and are summarized in Table 3. All admission variables were not found to predict NIV weaning duration. RR at the first wean, however, was a significant predictor of NIV weaning duration (*p* = 0.007). First wean RR equal to or greater than 40 breaths per minute (*n* = 45) had significantly longer weans than patients with less than 40 breaths per minute (17.9 versus 5.0 h; *p* = 0.007).

**TABLE 3:** Difference of means for NIV wean duration (h).

Variable	Total	Mean (h), SD	95% CI	*p* value
Comorbidities			−8.35 to 19.15	0.44
Yes	10	8.5 (13.9)		
No	64	13.3 (21.1)		
RSV status			−11.80 to 7.40	0.65
Yes	44	14.1 (11.9)		
No	30	20.5 (20.1)		
Sex			−111.29 to 8.09	0.74
Male	45	13.8 (12.2)		
Female	29	22.7 (16.2)		
Admission SpO_2_			−10.98 to 9.19	0.86
90–94	24	13.8 (12.9)		
95–100	50	20.1 (20.5)		
Admission RR			−2.64 to 16.04	0.16
< 40	39	10 (16.7)		
> 40	35	18.6 (21.7)		
First Wean RR			3.60 to 22.20	0.007
< 40	28	5 (17.9)		
> 40	45	11.2 (23)		

*Note:* BiPAP, bilevel positive airway pressure; FiO_2_, fraction of inspired oxygen; NIV, noninvasive ventilation; SpO_2_, peripheral oxygen saturation.

Abbreviations: CPAP, continuous positive airway pressure; RR, respiratory rate; SD, standard deviation.

Regarding outcomes, total PICU days were not significantly different between the CPAP and BiPAP groups (3.94 versus 5.11 days; *p* = 0.1887). Total hospital days were also not significantly different between the groups (5.77 versus 6.79 days; *p* = 0.1691). For all comers, our 7‐day readmission rate was 1.4%, and the 30‐day readmission rate was 2.7%.

## 4. Discussion

This retrospective cohort study evaluated variables related to NIV weaning for patients with bronchiolitis. The results highlighted the variability of weaning practices, exposed the notion of a complex wean, and introduced RR as a possible signal for NIV weaning readiness.

In our PICU, weaning practices for NIV were not uniform. Different strategies of weaning were shown by initial reductions of IPAP, EPAP, or both simultaneously. Some patients were even weaned off of NIV as the initial step. Decremental weans of IPAP and EPAP were also highly variable as suggested by their large standard deviations. However, without a standardized method or protocol, and despite instances of arguably overzealous weans, our patients with bronchiolitis were still weaned from NIV in less than 24 h. Although a standardized approach may have decreased the duration of the wean, the potential clinical advantages of this reduction would likely be minimal. Rather, a focus on when to begin the wean would likely lead to appreciably shortening of total NIV duration.

Our study found several children who were not weaned with a decremental stepwise approach. Rather, some were escalated and de‐escalated multiple times between settings and/or modalities. We labeled this observation as “complex weans,” and to our knowledge, it has not been described with bronchiolitis. In a study by Mortamet et al. [14], expert consensus labeled a successful wean off of NIV after 24–48 h with no respiratory support, and a weaning failure as a need to reinstall any type of respiratory support (invasive or noninvasive). No consensus was reached regarding the weaning procedure. The panel did not address various adjustments between settings and modalities during NIV weaning. In 2023, Cassiba et al. [8] employed a stepwise wean from BIPAP to CPAP to high‐flow nasal cannula and defined weaning failure as a switch back to previous modes of support. Similarly, a nurse‐driven protocol in 2025 used a stepwise approach to wean infants off CPAP, with infants who failed being escalated back to previous mode of support [12]. Escalation of settings and numerous switches between support modalities were not reported.

The concept of a complex wean was important in the current study as children on BiPAP were more likely to experience a complex wean, and children on BiPAP who were put on RA almost unanimously had a complex wean. The idea of a complex wean suggests that children may not have had an optimal wean, and this may be due to difficulty identifying patients who were ready to wean. Work of breathing may also play a role, as many children had their support escalated based on clinical work of breathing without changes in other clinical measures or vital signs. Identifying clear variables to initiate weaning of NIV, particularly for children on BiPAP, may help limit complex weans and appreciably shorten NIV duration.

Finally, admission variables of comorbidities, sex, RSV status, and admission vital signs were not found to predict NIV weaning duration. Importantly, this suggests that weaning timing depended less on admission severity and more on detecting a physiological signal that indicates a patient is ready to wean, regardless of their initial illness severity. Notably, RR was identified as a significant variable predicting duration of NIV wean. Specifically, a RR of 40 was shown to be a critical point after which weaning durations became longer. As minute ventilation is a product of RR and tidal volume, higher RR is necessary to compensate for decreased tidal volumes as seen with bronchiolitis. Consistent with this, RR has been shown as an important parameter for assessing NIV weaning readiness. Expert consensus has identified RR as an important clinical criterion for weaning NIV [14], with a RR of 60 being common for initiating weaning and identifying wean failure [10, 12]. In our study, children with higher RR had longer weaning times. Additionally, the children on BiPAP were weaned with higher RR and had more complex weans. Consistent with previous studies, our data support RR as a potential indicator of weaning readiness.

### 4.1. Limitations

This study had several limitations. First, tidal volume was not captured as a variable, highlighting the limitations of a retrospective study design. The absence of this variable may potentially influence the interpretation of RR findings. Second, bacterial infections have been shown in the literature to be possible predictors of NIV weaning failure [15]. Bacterial infections were recorded in the current study by means of urine and blood cultures, but bacterial pneumonia was diagnosed by chest X‐ray, making it difficult to draw conclusions about whether secondary bacterial infections influenced weaning duration or outcomes. First NIV weans may have included reductions in IPAP, EPAP, both, or weaning off NIV. It is possible that RR predicts weaning duration regardless of whether IPAP or EPAP is weaned first, but this could not be concluded in our data based on the variability of the first wean. The focus of this study was to identify a possible physiologic signal where children could be successfully weaned from NIV. Future research may help determine specific weaning strategies. Finally, admission data and NIV settings were collected from JPCH data when children were first admitted to our PICU to ensure consistency in data collection. However, vital signs or blood gas values prior to starting any form of NIV in referring centers could not be captured.

## 5. Conclusion

NIV has emerged as a therapeutic option for children with bronchiolitis admitted to the PICU. It follows that initiation must be matched with valid indicators for weaning and discontinuation. There is a paucity of guidelines that relate to pediatric NIV weaning protocols in bronchiolitis patients. The nonuniformity of current weaning practices was highlighted in our data and is consistent with previous literature identifying significant variability in current NIV weaning practices. RR was identified in our data as an important signal of NIV weaning readiness and efficiency. It may be an important clinical variable to consider in future weaning protocols. Finally, to the best of our knowledge, this is the first study to elucidate the notion of a complex wean, as an outcome measure in children with bronchiolitis on NIV support. Our results may be used to inform future guidelines for weaning NIV in children with bronchiolitis admitted to the PICU.

## Author Contributions

R.B. completed the literature review, data collection, and contributed to the writing and editing of the final manuscript. G.H. and T.H. coconceptualized the study, completed statistical analyses, and contributed to the writing and editing of the final manuscript.

## Funding

None of the authors have sources of financial support to declare.

## Disclosure

A preprint has previously been published [16]. This work was presented at Child Health Research Day, April 2025, Saskatoon SK, Canada, by Rheanna Bulten, and at the National Pediatric Resident Research Competition, May 2025, Quebec City, QB, Canada, by Rheanna Bulten.

## Conflicts of Interest

The authors declare no conflicts of interest.

## Data Availability

The data that support the findings of this study are available from the corresponding author upon reasonable request.
